# Evidence for altered host genetic factors in KSHV infection and KSHV‐related disease development

**DOI:** 10.1002/rmv.2160

**Published:** 2020-10-11

**Authors:** Melissa J. Blumenthal, Elena Maria Cornejo Castro, Denise Whitby, Arieh A. Katz, Georgia Schäfer

**Affiliations:** ^1^ International Centre for Genetic Engineering and Biotechnology Cape Town South Africa; ^2^ Division of Medical Biochemistry and Structural Biology, Department of Integrative Biomedical Sciences University of Cape Town Cape Town South Africa; ^3^ Institute of Infectious Disease and Molecular Medicine University of Cape Town Cape Town South Africa; ^4^ Viral Oncology Section, AIDS and Cancer Virus Program, Leidos Biomedical Research Frederick National Laboratory for Cancer Research Frederick Maryland USA

**Keywords:** AIDS‐related malignancy, candidate gene association, genetic susceptibility, human herpesvirus 8, Kaposi's sarcoma, Kaposi's sarcoma‐associated herpesvirus

## Abstract

Kaposi's sarcoma‐associated herpesvirus (KSHV) is the etiological agent of Kaposi's sarcoma (KS), the most common AIDS‐related malignancy. It also causes other rare, but certainly underreported, KSHV‐associated pathologies, namely primary effusion lymphoma, multicentric Castleman disease and KSHV inflammatory cytokine syndrome. Epidemiology and pathogenicity studies point to the potential for host genetic predisposition to KSHV infection and/or the subsequent development of KSHV‐associated pathologies partly explaining the peculiar geographic and population‐specific incidence of KSHV and associated pathologies and discrepancies in KSHV exposure and infection and KSHV infection and disease development. This review consolidates the current knowledge of host genetic factors involved in the KSHV‐driven pathogenesis. Studies reviewed here indicate a plausible connection between KSHV susceptibility and host genetic factors that affect either viral access to host cells via entry mechanisms or host innate immunity to viral infection. Subsequent to infection, KSHV‐associated pathogenesis, reviewed here primarily in the context of KS, is likely influenced by an orchestrated concert of innate immune system interactions, downstream inflammatory pathways and oncogenic mechanisms. The association studies reviewed here point to interesting candidate genes that may prove important in achieving a more nuanced understanding of the pathogenesis and therapeutic targeting of KSHV and associated diseases. Recent studies on host genetic factors suggest numerous candidate genes strongly associated with KSHV infection or subsequent disease development, particularly innate immune system mediators. Taken together, these contribute toward our understanding of the geographic prevalence and population susceptibility to KSHV and KSHV‐associated diseases.

**Abbreviations**
ART: antiretroviral therapy
CCL2: C‐C chemokine ligand 2
CCND1: cyclin D1
CDKN1A: cyclin dependent kinase inhibitor 1A
CFLAR: CASP8 and FADD‐like apoptosis regulator
EPHA2: Eph receptor A2
FcγR: Fc gamma receptors
IL8RB: IL8 receptor, beta
KICS: KSHV inflammatory cytokine syndrome
KIR: killer cell immunoglobulin‐like receptors
KS: Kaposi's sarcoma
KSHV: Kaposi's sarcoma‐associated herpesvirus
MBL: mannose‐binding lectin
MCD: multicentric Castleman disease
MDM2: mouse double minute 2
NFκB: nuclear factor kappa B
NFκBIA: NFκB1 inhibitor alpha
NK: natural killer
PEL: primary effusion lymphoma
PTEN: phosphatase and tensin homologue
SNP: single nucleotide polymorphism
SSA: Sub‐Saharan Africa
TLR4: toll‐like receptor 4
TP53: tumour protein 53
VEGF: vascular endothelial growth factor

## 1 INTRODUCTION

Kaposi's sarcoma‐associated herpesvirus (KSHV, or human herpesvirus‐8) is a gammaherpesvirus with a particularly high seroprevalence in sub‐Saharan Africa (SSA, 30%‐50%) and the Mediterranean region (20%‐30%).^1^, ^2^, ^3^ It is the etiological agent of the most common AIDS‐related malignancy, Kaposi's sarcoma (KS),^4^ as well as the rare (although most certainly under‐reported^5^) primary effusion lymphoma (PEL), multicentric Castleman disease (MCD) and KSHV inflammatory cytokine syndrome (KICS) which all primarily occur in HIV‐infected patients.^6^, ^7^, ^8^, ^9^, ^10^, ^11^ Exposure to and infection with KSHV is thought to occur early in life via saliva,^12^ whereupon the virus establishes long‐term persistent infection which is usually clinically silent even during intermittent lytic reactivation, likely controlled by T cell responses.^10^, ^13^, ^14^ However, with a decline in T cell immunity, most markedly in the context of HIV co‐infection but also in medically immunosuppressed and elderly people, KSHV‐infected patients become more likely to develop KSHV‐associated pathologies.^15^, ^16^, ^17^, ^18^

KSHV seroprevalence exhibits peculiar geographical epidemiology and incidence of KS and other KSHV‐associated diseases outside the setting of HIV are population‐specific.^3^, ^19^, ^20^ Furthermore, there are discrepancies in KSHV exposure vs infection and KSHV/HIV co‐infection vs KS development in addition to evidence of familial aggregation of KSHV seroprevalence.^12^, ^21^, ^22^ This growing body of evidence points to a potential underlying genetic risk of susceptibility to KSHV infection and associated disease development. In this review, we focus on studies reporting familial aggregation of susceptibility to KSHV infection and/or KSHV‐associated diseases and particular genetic polymorphisms in candidate susceptibility genes or haplotypes found to be statistically associated with aspects of KSHV pathogenesis. Further, we identify the limitations of gene association studies in isolation and suggest further research directions to elucidate the potential for targeted interventions in genetically susceptible populations.

## 2 EVIDENCE FOR THE EXISTENCE OF A GENETIC LINK

KSHV prevalence varies geographically and is particularly high in SSA (30%‐50%) and the Mediterranean region (20%‐30%).^1^, ^2^, ^3^ Higher prevalence has been noted in people of specific ethnicities regardless of HIV infection where KSHV (and KS) was endemic even before the HIV/AIDS epidemic, such as in Uganda (14%‐95%), the Ivory Coast (43%‐100%), Cameroon (≥80%), the Amazonian basin in Brazil (53%), Peru (56%) and among people of Uygur and Han ethnicity in Xinjiang, China (40%‐60% and 21%‐31%, respectively).^3^, ^20^, ^23^, ^24^, ^25^, ^26^, ^27^, ^28^ In HIV‐infected people in the USA on antiretroviral therapy (ART), prevalence was 38%.^29^ Furthermore, exposure to KSHV does not always result in KSHV infection; seroconversion even in areas of high exposure is 30% to 50%.^21^ An epidemiological population‐based study on 1337 individuals of African origin in French Guinea where KSHV is endemic, showed strong correlation of KSHV seroprevalence between mother‐child and sibling‐sibling pairs, suggestive of familial aggregation.^12^

KSHV infection is necessary for the development of KS, MCD, PEL and KICS but insufficient for tumorigenesis. Precipitating factors such as HIV infection or immune suppression are required for KSHV‐associated oncogenesis. Even so, HIV/KSHV co‐infection does not always result in cancer development. The most common of the KSHV‐associated pathologies, KS, is distinguished into four epidemiological forms. Classic KS primarily occurs in men from the Mediterranean and Eastern European region due to immune suppression related to old age, while endemic KS is found in Central Africa mostly in younger males.^17^, ^30^ Further, iatrogenic KS is associated with transplant‐related immunosuppression and therefore regresses with immune reconstitution.^31^, ^32^ With the onset of the AIDS epidemic, the incidence of an AIDS‐related or epidemic KS form, the most clinically aggressive, has burgeoned, driven by HIV/AIDS‐related immune suppression and HIV itself.^33^, ^34^ Within these epidemiological groupings, KS occurs with specific geographic distribution.^19^ Risk of classic KS is threefold higher in men from the South of Italy than the North.^35^ Transplant recipients of Mediterranean, Jewish and Middle Eastern ancestry (at risk for classic KS) are also overrepresented among iatrogenic KS patients regardless of their residence at the time of the transplant.^19^, ^35^, ^36^ Among HIV‐positive patients, risk of developing AIDS‐KS is reported to be higher in South Africa than in Europe, North America and Latin America and the trend of decreased risk concomitant with increased CD4 count after initiation of ART evident in these regions was not observed in South Africa.^22^ Geographic and population‐specific prevalence of KSHV and incidence of KS suggest a potential role for host genetic factors in susceptibility to KSHV infection and subsequent development of KS. Similarly, African ancestry has been shown to be a significant independent predictor of MCD incidence in HIV patients, suggesting a putative genetic susceptibility.^37^

These observations point to genetic susceptibility to KSHV infection following exposure and/or progression to KS or other KSHV‐associated pathologies.^3^, ^38^, ^39^, ^40^

## 3 CANDIDATE SUSCEPTIBILITY GENES—CURRENT KNOWLEDGE

Thus far, immune‐modulatory genes have been the focus of investigations of candidate susceptibility genes. In particular, published studies have reported significant associations of KSHV infection or its associated pathologies with genetic polymorphism in genes encoding interleukins *IL6*, *IL8* and *IL13*,^41^, ^42^, ^43^, ^44^ vascular endothelial growth factor (*VEGF*),^45^ nuclear factor kappa B (*NFκB*),^46^ mannose‐binding lectin (*MBL*)‐2,^47^ Fc gamma receptors (*FcγR*),^48^ *HLA* killer cell immunoglobulin‐like receptors (*KIR*)^49^, ^50^ and their *HLA* ligands and linked genes^50^, ^51^, ^52^, ^53^, ^54^, ^55^, ^56^ and homologues of human genes pirated by KSHV, namely cyclin D1 (*CCND1*), *IL6*, C‐C chemokine ligand 2 (*CCL2*) and FADD‐like apoptosis regulator (*CFLAR*).^57^ Additionally, we identified genetic variants in the KSHV entry receptor Eph receptor A2 (*EPHA2*) to be significantly associated with KSHV infection and KS development.^58^ Conversely, tumour necrosis factors (*TNF*)*‐α* and *‐β*,^41^, ^44^ *IFNγ*,^44^ stromal‐derived factor 1 (*SDF1*), cyclin dependent kinase inhibitor 1A (*CDKN1A*),^59^ C‐C chemokine receptor type 5 (*CCR5*)^41^ and caspase 8 (*CASP8*)^57^ have been investigated in relation to KS and KSHV but have not yielded statistically significant results. The above‐mentioned studies reporting statistically significant associations of host genetic polymorphisms or haplotypes with KSHV or KSHV‐associated pathologies are summarised in Table 1.

**TABLE 1:** Summary of sequence variants and haplotypes associated with susceptibility to KSHV or KSHV‐associated pathologies

Gene	HGVS^a^ sequence variant (rsid)/ allele/ haplotype	OR (95% CI)	*P* value	Associated with	Description of study cohort	Cases vs controls	Reference
*Susceptibility to KSHV infection*							
*VEGF*	NG_008732.1:g.4822C>A (rs59260042)	4.8 (1.4‐17.1)	.005	KSHV viral load	Renal transplant recipients, KSHV+ or KSHV− after transplant	44 vs 128	Alkharsah et al^45^
	NM_001025366.3:c.‐94C>T (rs2010963)	3.98 (1.5‐11.1)	.004	KSHV viral load	Female only renal transplant recipients, KSHV+ or KSHV− after transplant	18 vs 50	Alkharsah et al^45^
*NFκB1*	NG_050628.1:g.4670_4673ATTG[1] (rs28362491)	7.9 (3.3‐19.1)	<.001	KSHV lytic antibody response	HIV+/KSHV+ patients	63 vs 69	Gonçales et al^46^
*NFκBIA*	NM_020529.3:c.*126G>A (rs696)	Het: 12.3 (4.3‐34.9) Hom: 9.4 (3.2‐27.9)	<.001	KSHV lytic antibody response	HIV+/KSHV+ patients	63 vs 69	Gonçales et al^46^
*MBL2*	HYA/HXA, HYA/ HYO, HYA/LXA, HYA/LYO, LXA/LXA, LYA/LXA and LYA/LYO	3.1 (1.2‐7.6)	.02	CD4 count response	HIV+ patients who were KSHV+ or KSHV−	124 vs 213	De Morais et al^47^
*HLA* and *KIR*	*HLA‐B Bw4‐80I*+ and *KIR3DS1*	0.6 (0.4‐0.9)	.01	KSHV seroprevalence	HIV−/KSHV+ or KSHV− patients	277 vs 562	Goedert et al^50^
*HLA* class I	HLA‐C*01, *03, *07, *08	0.6 (0.4–0.9)	.01	KSHV seroprevalence	HIV−/KSHV+ or KSHV− patients	272 vs 549	Goedert et al^50^
	HLA‐A*6801	3.5 (1.4‐8.5)	.0045	KSHV shedding in saliva	HIV− mothers with or without detectable KSHV in saliva	84 vs 241	Alkharsah et al^60^
	HLA‐A*4301	5.3 (1.7‐17)	.002	KSHV shedding in saliva	HIV− mothers with or without detectable KSHV in saliva	84 vs 241	Alkharsah et al^60^
*HLA* class II	HLA‐DRB1*04	4.1 (1.4‐11.5)	.0067	KSHV shedding in saliva	Mothers with or without detectable KSHV in saliva	31 vs 332	Alkharsah et al^60^
*EPHA2*	NM_004431.5:c.2572C>T	6.4 (1.4‐28.4)	.03	KSHV serumpositivity	HIV+/KSHV+ or HIV+/KSHV− patients	100 vs 50	Blumenthal et al^58^
*Susceptibility to KSHV‐associated pathologies*							
*IL6*	XM_011515390.2:c.‐84‐153C>G (rs1800795)	2.11 (1.2‐3.7)	.0046	AIDS‐related KS	HIV+ male patients with or without KS	115 vs 126	Foster et al^41^
		5.3 (1.5‐18.9)	.008	Iatrogenic KS	Renal transplant recipients with or without KS	15 vs 40	Gazouli et al^42^
		ns	ns	Classic KS	HIV−/KSHV+ patients with or without KS	132 vs 169	Brown et al^44^
*IL8*	NM_000584.4:c.65‐204C>T (rs2227306)	0.62 (0.38‐1.00)	.16	Classic KS	HIV−/KSHV+ patients with or without KS	132 vs 167	Brown et al^44^
	NG_029889.1:g.4802A>T (rs4073)	ns	ns	Classic KS	HIV−/KSHV+ patients with or without KS	132 vs 164	Brown et al^44^
		0.49 (0.25‐0.97)	.039	AIDS‐related KS	HIV+/KSHV+ male patients with or without KS	84 vs 154	van der Kuyl et al^43^
*IL8RB*	NM_001557.4:c.*127T>C (rs1126579)	Het: 0.51 (0.31‐0.84) Hom: 0.45 (0.21‐0.96)	.01	Classic KS	HIV−/KSHV+ patients with or without KS	133 vs 167	Brown et al^44^
	NM_001557.4:c.*359G>A (rs1126580)	0.48 (0.24‐0.94)	.07	Classic KS	HIV−/KSHV+ patients with or without KS	129 vs 160	Brown et al^44^
*IL13*	NM_002188.3:c.431A>G (rs20541)	1.82 (1.12‐2.97)	.04	Classic KS	HIV−/KSHV+ patients with or without KS	133 vs 168	Brown et al^44^
*FcγRIIIA*	NM_000569.8:c.526T>C (rs396991)	2.47 (1.46‐4.16)	.0063	AIDS‐related KS	HIV‐infected males with or without KS	112 vs 128	Lehrnbecher et al^48^
*HLA* and *KIR*	*HLA‐B Bw4‐80I*+ and *KIR3DS1*	2.1 (1.3‐3.4)	.002	Classic KS	HIV−/KSHV+ patients with or without KS	248 vs 277	Goedert et al^50^
		2.06 (0.4‐12.3)	ns	Classic KS	Patients with or without KS (those without KS were KSHV+ or KSHV−)	11 vs 19	Guerini et al^49^
		ns	ns	AIDS‐related KS	HIV‐infected patients with or without KS	81 vs 88	Qi et al^61^
	*HLA‐B Bw4‐80I*‐ and *KIR3DS1*	6.00 (1.5‐24.6)	.006	Classic KS	Patients with or without KS (those without KS were KSHV+ or KSHV−)	21 vs 32	Guerini et al^49^
*KIR*	*KIR2DS1*	3.82 (1.4‐10.9)	.008	Classic KS	Patients with or without KS (those without KS were KSHV+ or KSHV−)	32 vs 51	Guerini et al^49^
	*KIR3DS1*	4.0 (1.4‐11.4)	.006	Classic KS	Patients with or without KS (those without KS were KSHV+ or KSHV−)	32 vs 51	Guerini et al^49^
*HLA* class I	HLA‐A*11:*01*	0.4 (0.2‐0.7)	.002	Classic KS	HIV− patients with or without KS	248 vs 855	Goedert et al^50^
	HLA‐C*01, *03, *07, *08	1.8 (1.2‐2.7)	.005	Classic KS	HIV− patients with or without KS	247 vs 272	Goedert et al^50^
	HLA‐C*07:*01*	1.6 (1.2‐2.1)	.002	Classic KS	HIV− patients with or without KS	250 vs 846	Goedert et al^50^
	HLA‐B*14:*01*	4.2 (1.1‐15.5)	.03	AIDS‐related KS	HIV+/KSHV+ patients with and without KS	348 vs 318	Aissani et al^51^
		4.27 (1.67‐10.91)	.033	AIDS‐related KS	HIV+ patients with or without KS	157 vs 523	Cornejo Castro et al^52^
	HLA‐B*14	0.14 (0.02‐0.7)	.0135	AIDS‐related KS	HIV+ patients with or without KS	116 vs 59	Marmor et al^62^
	HLA‐B*2702/*5*	0.37 (0.15‐0.94)	.04	AIDS‐related KS	HIV+/KSHV+ patients with and without KS	348 vs 318	Aissani et al^51^
		0.39 (0.16‐0.94)	.04	AIDS‐related KS	HIV+ CD4 decline matched patients with or without KS	96 vs 96	Dorak et al^53^
	*HLA‐CW4*	4.96 (2.9‐8.12)	.03	Iatrogenic KS	HIV− renal transplant recipients with or without KS	44 vs 15	Azmandian et al^54^
	*HLA‐A30*	0.48 (0.25‐0.90)	.48	Classic KS	HIV− patients with or without KS	62 vs 220	Masala et al^55^
	*HLA‐CW5*	0..32 (0.16‐0.65)	.0006	Classic KS	HIV− patients with or without KS	62 vs 220	Masala et al^55^
	*HLA‐CW7*	2.48 (1.27‐4.72)	.01	Classic KS	HIV− patients with or without KS	62 vs 220	Masala et al^55^
	*HLA‐B58*	0.035 (0.002‐0.58)	.00001	Classic KS	HIV− patients with or without KS	62 vs 220	Masala et al^55^
*HLA* class II	HLA‐DRB1*1302‐DQB1*0604	6.12 (1.29‐28.9)	.02	AIDS‐related KS	HIV+, CD4 decline matched patients with or without KS	96 vs 96	Dorak et al^53^
	HLA‐DRB1*F13	2.24 (1.19‐4.20)	.016	AIDS‐related KS	AIDS patients with or without KS	122 vs 94	Gaya et al^56^
	HLA‐DRB1*1104	2.12 (1.05‐4.25)	.047	Classic KS	HIV− patients with or without KS	62 vs 220	Masala et al^55^
	HLA‐DRB1*1302	5.83 (1.73‐19.83)	.004	Classic KS	HIV− patients with or without KS	62 vs 220	Masala et al^55^
	HLA‐DRB1*1601	0.50 (0.26‐1.0)	.043	Classic KS	HIV− patients with or without KS	62 vs 220	Masala et al^55^
	HLA‐DQA1*0302	11.97 (1.27‐103.36)	.019	Classic KS	HIV− patients with or without KS	62 vs 220	Masala et al^55^
	HLA‐DQB1*0502	0.52 (0.27‐0.97)	.047	Classic KS	HIV− patients with or without KS	62 vs 220	Masala et al^55^
	HLA‐DQB1*0604	7.75 (2.02‐29.70)	.0017	Classic KS	HIV− patients with or without KS	62 vs 220	Masala et al^55^
*HLA‐DMB*	NM_002118.4:c.55+649T>C (rs6902982)	4.09 (1.90‐8.80)	.0003	AIDS‐related KS	HIV+/KSHV+ patients with and without KS	348 vs 318	Aissani et al^51^
*TAP1*	NM_000593.5:c.2090A>G (rs1135216)	1.54 (1.09‐2.18)	.014	AIDS‐related KS	HIV+/KSHV+ patients with and without KS	348 vs 318	Aissani et al^51^
	NM_000593.5:c.1177A>G (rs1057141)	1.45 (1.05‐1.99)	.024	AIDS‐related KS	HIV+/KSHV+ patients with and without KS	348 vs 318	Aissani et al^51^
*TAP1/TAPSAR1*	NG_011759.1:g.13891T>C (rs2071541)	1.6 (1.11‐2.32)	.012	AIDS‐related KS	HIV+/KSHV+ patients with and without KS	348 vs 318	Aissani et al^51^
*GPANK1*	NM_001199237.1:c.*90T>C (rs7029)	1.55 (1.17‐2.05)	.002	AIDS‐related KS	HIV+/KSHV+ patients with and without KS	348 vs 318	Aissani et al^51^
*TRIM31*	NM_007028.3:c.1261G>A (rs1116221)	0.74 (0.56‐0.96)	.033	AIDS‐related KS	HIV+/KSHV+ patients with and without KS	348 vs 318	Aissani et al^51^
*LTA*	NM_000595.4:c.‐10+90A>G (rs909253)	0.75 (0.58‐0.96)	.022	AIDS‐related KS	HIV+/KSHV+ patients with and without KS	348 vs 318	Aissani et al^51^
*LY6G6C*	NM_025261.3:c.243C>T (rs1065356)	1.60 (1.18‐2.16)	.002	AIDS‐related KS	HIV+/KSHV+ patients with and without KS	348 vs 318	Aissani et al^51^
*EPHA2*	NM_004431.5:c.2099T>C NM_004431.5:c.2835G>T	1.2 (1.1‐1.3) 1.2 (1.1‐1.4)	.04 .02	AIDS‐related KS	HIV+/KSHV+ patients with or without KS	50 vs 50	Blumenthal et al^58^
*TLR4*	NM_138554.5:c.896A>G (rs4986790)	4.34 (1.3‐14.4)	.021	MCD	HIV+ patients with or without MCD	20 vs 89	Lagos et al^63^

^a^ Variants are named according to Human Genome Variation Society (HGVS)‐nomenclature^64^ with reference to their reference SNP identification (rsid) number, corresponding to the SNP database, where applicable. Alternatively, the haplotype is given where appropriate. HLA haplotypes are named according to the naming convention determined by the WHO Nomenclature Committee for Factors of the HLA System. Odds ratio (OR), confidence interval (CI) and *P* values are extracted from papers referenced in the table or calculated from the published data as required. An OR >1 is indicative of increased risk; OR <1 indicates decreased risk; ns indicates an OR with a confidence interval crossing 0 and a *P* value >.05.

### 3.1 Susceptibility to KSHV infection

A foundational study conducted by Plancoulaine et al^65^ identified, by segregation analysis of KSHV seroprevalence among a French Guinean population, the presence of an unidentified recessive major gene that affects, in combination with age, KSHV seroconversion in children under 10 years of age. This was mapped to chromosome region 3p22 which encodes the following genes: programmed cell death 6 interacting protein (*PDCD6IP*), ubiquitin‐specific protease (*UBP*), F‐box and leucine‐rich repeat protein (*FBXL2*), cyclic AMP‐regulated phosphoprotein 21 (*ARPP‐21*), leucine‐rich repeat flightless‐interacting protein 2 (*LRRFIP2*) and C‐C motif chemokine receptor 4 (*CCR4*).^66^ Several studies assessing genetic factors underlying susceptibility to KSHV infection have since been conducted.

Two triplet infants have been reported with familial hemophagocytic lymphohistiocytosis related to compound heterozygous perforin mutations and KSHV infection; in these cases, a defective perforin‐granzyme pathway is suggested to have led to reduced viral clearance ability and therefore to the onset of symptoms related to lytic KSHV infection.^67^ Other studies have similarly focused on viral immune response genes. *MBL2* haplotypes (based on combinations of two promoter region genotypes (−550 H/L and −221 Y/X) and a structural region genotype (exon 1 A/O)) associated with intermediate expression of MBL, an innate immune system protein, were found to be associated with lower CD4 count in HIV and KSHV co‐infected patients compared to patients with only HIV infection.^47^ Additionally, polymorphisms in the *NFκB1* promoter (NG_050628.1:g.4670_4673ATTG[1], reference SNP (rs)28 362 491) and the 3′ UTR region of the *NFκB1* inhibitor alpha (NM_020529.3:NFκBIA c.*126G>A, rs696) were found to be associated with the presence of antibodies to KSHV lytic antigens.^46^ Natural killer (NK) cell activation is important in the immune response to viruses and proposed to be protective against KSHV infection.^50^, ^68^ NK cells are regulated by killer Ig‐like receptors (KIR) which are activated by interaction with HLA molecules. The combination of *KIR3DS1* (an activating haplotype of *KIR*) and *HLA‐B Bw4‐80I* (the *Bw4* haplotype with an isoleucine at position 80) as well as *HLA‐C* group 1 (HLA‐C*01, *03, *07, *08) homozygosity was indeed found to be protective against KSHV infection.^50^ Conversely, HLA‐A*6801, HLA‐A*4301 and HLA‐DRB1*04 were associated with KSHV shedding in saliva in mothers in rural South Africa,^60^ while HLA‐DRB1 alleles investigated in relation to KSHV susceptibility in a Sardinian population were not shown to be statistically associated although HLA‐DR2 antigens were slightly overrepresented in KSHV‐positive patients (but did not withstand statistical correction for multiple testing).^69^

Key molecules involved in the initial stages of KSHV entry are interesting candidate genes for KSHV association studies. We investigated sequence variants in a major KSHV entry receptor in endothelial cells, *EPHA2*, in relation to KSHV infection. Mutation analysis revealed a novel heterozygous transition (NM_004431.5:c.2572C>T) associated with KSHV infection in a cohort of HIV‐infected South African patients stratified by KSHV status.^58^ The angiogenesis‐related *VEGF*, shown to augment entry of KSHV into target cells,^70^ harboured a promoter region SNP (NG_008732.1:g.4822C>A, rs59260042) associated with KSHV viremia in kidney transplant recipients, and a SNP in the 5′ untranslated region (UTR, NM_001025366.3:c.‐94C>T, rs2010963) was likewise associated with KSHV viremia but in females only.^45^

As the necessary etiological agent for the development of KS, MCD, PEL and KICS, enhanced susceptibility to KSHV infection subsequently increases the risk for KSHV‐associated pathologies. However, KSHV‐associated tumorigenesis may be caused by additional underlying genetic factors as described below.

### 3.2 Susceptibility to KSHV‐associated pathologies

As the most prevalent of the KSHV‐associated pathologies, KS, also the most common AIDS‐related malignancy worldwide, has been the focus of most studies concerning genetic susceptibility to KSHV‐associated pathology. Other KSHV‐associated pathologies, namely MCD, PEL and KICS, are considered rare although are almost certainly underreported due to difficult diagnoses, particularly in resource‐limited regions where HIV burden is high. Consequently, there is a lack of research into the rarer KSHV‐associated pathologies and particularly on potential host genetic contributors; therefore, only few reports were identified for inclusion in this review.

#### 3.2.1 Kaposi's sarcoma

There is evidence from several case reports to suggest that genetic primary immunodeficiency predisposes to KS.^71^, ^72^, ^73^, ^74^, ^75^ Three unrelated Turkish children, aged 2, 9 and 9, born to consanguineous parents were reported to develop classic KS in early childhood, an extremely rare occurrence in the Mediterranean basin, strongly suggesting autosomal recessive predisposition.^73^ One of these children, who died from severe disseminated KS at the age of two, harboured a homozygous splice‐site stromal interaction molecule 1 (*STIM1*) mutation resulting in T cell immunodeficiency.^74^ A further case report describes a Turkish boy with inherited IFNγR1 deficiency due to homozygous *INFGR1* mutation (NP_000407.1:p.Cys77Tyr, rs104893974) who developed and died from classic KS.^71^ A cryptic splicing mediated *CTLA4* haploinsufficiency, associated with defective NK cell function, has recently been shown to have facilitated classic KS development in the case of a 52‐year‐old HIV‐negative male of Italian ancestry.^75^ A Tunisian boy with Wiskott‐Aldrich syndrome caused by a *WAS* deletion (NP_000368.1:p.Asp130_Glu131del) provides further evidence of a possible predisposition to classic KS.^72^

Several notable associations have been identified in genes encoding proteins that are known to be pro‐inflammatory. A SNP in the *IL6* promoter (XM_011515390.2:c.‐84‐153C>G, rs1800795) which is associated with increased levels of IL6 was noted in a familial clustering of classic KS and was further associated with KS in HIV‐infected men and renal transplant recipients.^39^, ^41^, ^42^ IL6 potently promotes the growth of KS spindle cells in an autocrine and paracrine manner.^76^ Additionally, a SNP in the *IL13* promoter region (NM_002188.3:c.431A>G, rs20541) was associated with classic KS in patients latently infected with KSHV.^44^ Similarly, an *IL8* promoter SNP (NG_029889.1:g.4802A>T, rs4073), linked to below normal IL8 expression, was identified in a cohort of patients with classic KS^44^; however, it was conversely found to decrease the risk of AIDS‐KS in HIV‐positive patients.^43^ An *IL8* SNP (NM_000584.4:c.65‐204C>T, rs2227306) was reported to decrease risk of classic KS^44^ and the combination of two SNPs (NM_001557.4:c.*127T>C, rs1126579 and NM_001557.4:c.*359G>A, rs1126580) in the human homologue to the KSHV‐encoded viral G‐protein coupled receptor (*vGPCR*), *IL8* receptor beta (*IL8RB*), were similarly found to be protective against the development of classic KS.^44^

Immunoregulatory genes have also been implicated in KS development in addition to KSHV susceptibility. A polymorphic form (NM_000569.8:c.526T>C, rs396991) of the IgG binding receptor, *FcγRIIIA*, was associated with AIDS‐KS in a cohort of HIV‐positive men and was found to enhance IgG affinity in vitro and promote NK cell activation.^48^, ^77^ Inflammation mediated by NK cell activation is postulated to promote KS oncogenesis.^50^, ^68^ Goedert et al^50^ reported that the combination of *KIR3DS1* (an activating haplotype of *KIR*) and *HLA‐B Bw4‐80I* (the *Bw4* haplotype with an isoleucine at position 80) was protective against KSHV seroprevalence (see Section 3.1) but increased the risk of classic KS among KSHV‐positive patients in a cohort of HIV‐negative patients without KS, however, this association was not corroborated in a HIV‐infected cohort investigated for KS development^61^ nor a smaller cohort of Italian classic KS patients, in which the *KIR3DS1*+/Bw4*80I‐genotype was statistically more frequent among classic KS cases than control.^49^ Additionally, activating *KIR* haplotypes, *KIR3DS1* and *KIR2DS1*, were associated with classic KS.^49^ Similarly, Goedert et al^50^ found *HLA‐C* group 1 (HLA‐C*01, *03, *07, *08) homozygosity to be protective against KSHV infection (see Section 3.1), but once infected to confer increased risk of KS development through a proposed mechanism of NK cell activation and induced inflammation.

Additionally, several *HLA* class I and II haplotypes have been thoroughly investigated. KS development post kidney transplant was associated with *HLA‐CW4* in a small study.^54^ In a familial clustering of classic KS, the homozygous HLA‐A*24/B*18/Cw*12/DRB1*11/DQB1*03 haplotype was present in family members with KS and not in those without KS, with the DRB1*11 subtype present in most family members.^39^ In a study of classic KS in a Sardinian population (high risk for classic KS), a number of class I and class II *HLA* haplotypes were associated with an increased (class I: *HLA‐CW7*; Class II: HLA‐DRB1*1104, HLA‐DRB1*1302, HLA‐DQA1*0302, HLA‐DQB1*0604) or decreased (class I: *HLA‐A30*, *HLA‐CW5*, *HLA‐B58*; class II: HLA‐DRB1*1601, HLA‐DQB1*0502) risk of classic KS.^55^ HLA‐C*07:*01* has also been associated with classic KS, while HLA‐A*11:*01* was found to decrease risk^50^ and HLA‐B*2705 was similarly reported to be protective.^51^, ^53^ *HLA‐B14* has been reported to be underrepresented among AIDS‐KS cases,^62^ but the specific allele, HLA‐B*14:*01*, was found to be a risk allele for AIDS‐related KS.^51^, ^52^ HLA‐DRB1*1302 in linkage disequilibrium with DQB1*0604 was identified as a risk haplotype for AIDS‐related KS^53^ as were *HLA‐DRB1* alleles with a phenylalanine residue at position 13.^56^ *HLA‐DR5* has been noted to be present at an increased frequency among (presumed) AIDS‐related KS,^78^ classic KS patients^78^, ^79^ as well as among iatrogenic KS patients^80^, ^81^, ^82^ while other studies have not found this to be the case.^62^

Several variants identified in a SNP screening of the *HLA‐DMB* gene region were found to increase AIDS‐KS risk. Most significantly, a SNP in the *HLA‐DMB* intronic region (NM_002118.4:c.55+649T>C, rs6902982) was associated with a four‐times higher risk of AIDS‐KS in HIV‐KSHV co‐infected men.^51^ Non‐synonymous SNPs in tripartite motif‐containing protein 31 (*TRIM31*, NM_007028.3:c.1261G>A, rs1116221) and lymphotoxin alpha (*LTA*, NM_000595.4:c.‐10+90A>G, rs909253) were observed to be protective whereas SNPs within *HLA‐DMB* linked genes, transporter associated with antigen processing 1 (*TAP1*, NM_000593.5:c.2090A>G, rs1135216; c.1177A>G, rs1057141), *TAPSAR1* microRNA (NG_011759.1:g.13891T>C, rs2071541), G‐patch domain and ankyrin repeats 1 (*GPANK*, NM_001199237.1:c.*90T>C, rs7029) and lymphocyte antigen 6 family member G6C (*LY6G6C*, NM_025261.3:c.243C>T, rs1065356), were associated with increased risk of AIDS‐KS.^51^

Recently, human genes that are cellular homologues of KSHV‐expressed genes have been investigated hypothesising that virally expressed homologues, which promote immune‐silent proliferation, may be advantaged by genetic SNPs in their cellular counterparts.^57^ A SNP screening in AIDS‐KS patients including several cellular homologues identified SNPs in *CCND1*, *IL6*, *CCL2* and *CFLAR* (homologues of KSHV‐encoded *vCyclin*, *vIL6*, viral FLICE‐inhibitory protein (*vFLIP*) and viral CC chemokine ligand (*vCCL*), respectively). Various combinations of these SNPs (but not a single SNP alone) were associated with AIDS‐KS.^57^

EPHA2, in addition to being a key entry receptor for KSHV into endothelial cells, has been implicated in oncogenesis via alteration of its canonical signalling pathway and noncanonical signalling pathway mediated by phosphorylation of key tyrosine and serine residues.^83^, ^84^ Indeed, mutation analysis revealed two novel, non‐synonymous heterozygous variants (NM_004431.5:c.2099T>C and NM_004431.5:c.2835G>T) in the *EPHA2* tyrosine kinase domain to be significantly associated with KS in a cohort of HIV‐infected South African patients stratified by KS status.^58^

#### 3.2.2 Other KSHV‐associated pathologies

In addition to KS, rare (although likely underreported) KSHV‐associated pathologies MCD, PEL and KICS may develop in KSHV‐infected patients, particularly in the context of KSHV/HIV co‐infection.

Childhood MCD, like classic KS, may be favoured by rare inborn errors of immunity against KSHV infection as suggested by Leroy et al^85^ in their report of a Comorian child born to consanguineous parents who presented with MCD at the age of 7. However, a study from Japan including 342 MCD patients found no evidence of family history.^86^ A toll‐like receptor 4 (*TLR4*) SNP (NM_138554.5:c.896A>G, rs4986790) which results in decreased surface expression of TLR4, an important pattern recognition receptor in activation of the innate immune system, was associated with increased incidence of MCD in KSHV+ HIV‐1+ patients compared to non‐KSHV cancer controls and patients with KS^63^; this SNP was further linked to patients with African ancestry.^87^

KICS and PEL, under‐researched in themselves, are yet to be investigated in relation to genetic associations.

## 4 LIMITATIONS OF CURRENT REPORTS AND GENETIC ASSOCIATION STUDIES

The major limitation of genetic association studies, including those reported in this review, is lack of consistent replication due to the small number of studies conducted and difficulties related to case‐control association studies. Indeed, few associations reported here have been satisfactorily replicated yet, with the exception of the reported association of an *IL6* promoter polymorphism (XM_011515390.2:c.‐84‐153C>G) with increased risk of AIDS‐related KS and iatrogenic KS,^41^, ^42^ the HLA‐B*1401 allele with increased risk of AIDS‐KS^51^, ^52^ and the HLA‐B*2702/*5* haplotype with decreased risk of AIDS‐related KS.^51^, ^53^

Observed associations may well be ethnicity‐ and geography‐specific, especially with regards to HLA haplotypes, as allele frequencies vary and as such may not be able to be replicated in studies conducted in different populations. Even so, replicability is hindered by heterogeneous study populations, with proposed associated genetic polymorphisms potentially being artefacts of population stratification or other confounding factors between cases and controls.^88^ Some populations included in studies reviewed here are ethnically diverse, particularly recently admixed populations, such as those from Brazil,^46^, ^47^ South Africa^58^ and the United States.^51^ Family studies, such as the report by Guttman‐Yassky et al^39^ of the *IL6* promoter GC genotype and the HLA‐DRB1*11 allele in a familial clustering of classic KS, are thought to bypass this limitation although these are limited in population applicability and statistical power and also require validation.

A narrowly defined phenotype to test in candidate association studies is important for consistent results. Lagging diagnostics, particularly of KSHV‐associated pathologies other than KS, are an issue. Even diagnosis of KSHV infection (by antibody response using various serological assays^23^, ^46^, ^58^ or viral load in peripheral blood leukocytes^45^) or KS (by clinical examination with or without indicated biopsy and histological confirmation,^50^, ^52^, ^58^ with or without investigations for visceral KS and with or without tumour staging) is heterogeneously performed or not reported at all. For example, KSHV seropositivity may not be a consistently defined phenotype between studies, as Newton et al^23^ noted disparity in KSHV seroprevalence estimates between their study and those previously reported due to the sensitivity of different serological assays (peptide based EIAs, protein ELISA and immunofluorescent assays). This highlights an underlying disjunction between the clinical and laboratory aspects of research. Clear and consistent diagnostic frameworks and phenotype standardization implemented in the clinical research setting is needed to overcome this in future studies. Further variability is introduced through differing laboratory techniques used to identify genetic polymorphism (eg, SNP screening using a commercial BeadArray genotyping platform of selected SNPs from candidate genes^51^ vs Sanger sequencing of the entire coding region of candidate genes with a discovery approach^58^), and future research would benefit from established guidelines to promote consistency. For instance, the numerous studies investigating *HLA* haplotypes reported here are certainly indicative of an association with KS, particularly in class I and II *HLA* types. However, the heterogeneity of studied populations and different *HLA* genotyping techniques (eg, targeted next generation sequencing method^52^ vs PCR‐sequence based typing^50^) used has led to inconsistent reports that require validation.^52^ Careful attention should be paid to the design of future studies to ensure validity and reproducibility.

## 5 POTENTIAL TARGETS FOR FUTURE INVESTIGATION

While a significant amount of research has been conducted into genetic polymorphisms associated with KS development, it would be beneficial for future research to address the possibility of a host genetic contribution to a broader range of KSHV‐associated pathologies. As the prerequisite for all KSHV‐related disease developments, emphasis should be placed on understanding susceptibility to KSHV infection. KSHV entry (most recently reviewed by Kumar and Chandran^89^ and Dollery^90^) is therefore an interesting target for study, and recent advances in understanding these mechanisms offer many new candidate susceptibility genes for investigation. Further insights into KSHV infection mechanisms provide new potential candidate genes such as the androgen receptor, recently described by Wang et al,^91^ to promote KSHV endocytosis and trafficking via EPHA2 serine phosphorylation.

While classic *p53* mutations have not been implicated in KSHV‐associated pathologies, *p53*‐linked genes are under‐researched. A plausible candidate SNP for future studies is a homozygous variant (309 G/G) of the *p53*‐modulator, *MDM2*, which has been suggested as a potential risk factor for development of visceral KS, PEL or MCD after the heterozygous form was found to be associated with cutaneous classic KS in Caucasians and the homozygous form identified in a number of PEL cell lines.^59^, ^92^

Improved understanding of specific genes involved in KSHV infection and KSHV‐related disease causation may have implications in the development of new therapeutic targets, and candidate gene association studies are therefore a relevant avenue of research. Although many different genetic variants predisposing individuals to either KSHV infection and/or KSHV‐associated diseases appear to exist which makes designing a therapy against all of them rather difficult, there are a few promising candidate genes: familiar KS has been linked to inborn defects in T cell immunity, in particular T cell effector functions and possibly interferon signalling,^71^, ^74^ while genetic polymorphism associated with differing sensitivities to cytotoxic drugs have been reported, such as the EPHA2 substitution, NP_004422.2:p.Gly391Arg, which showed increased sensitivity to rapamycin^93^, ^94^ and low dose resistance to cisplatin^94^ in human lung epithelial cells. Selected validated genetic variants conferring KSHV and KS susceptibility should therefore be assessed for drug sensitives.

## 6 CONCLUSIONS

**FIGURE 1:**
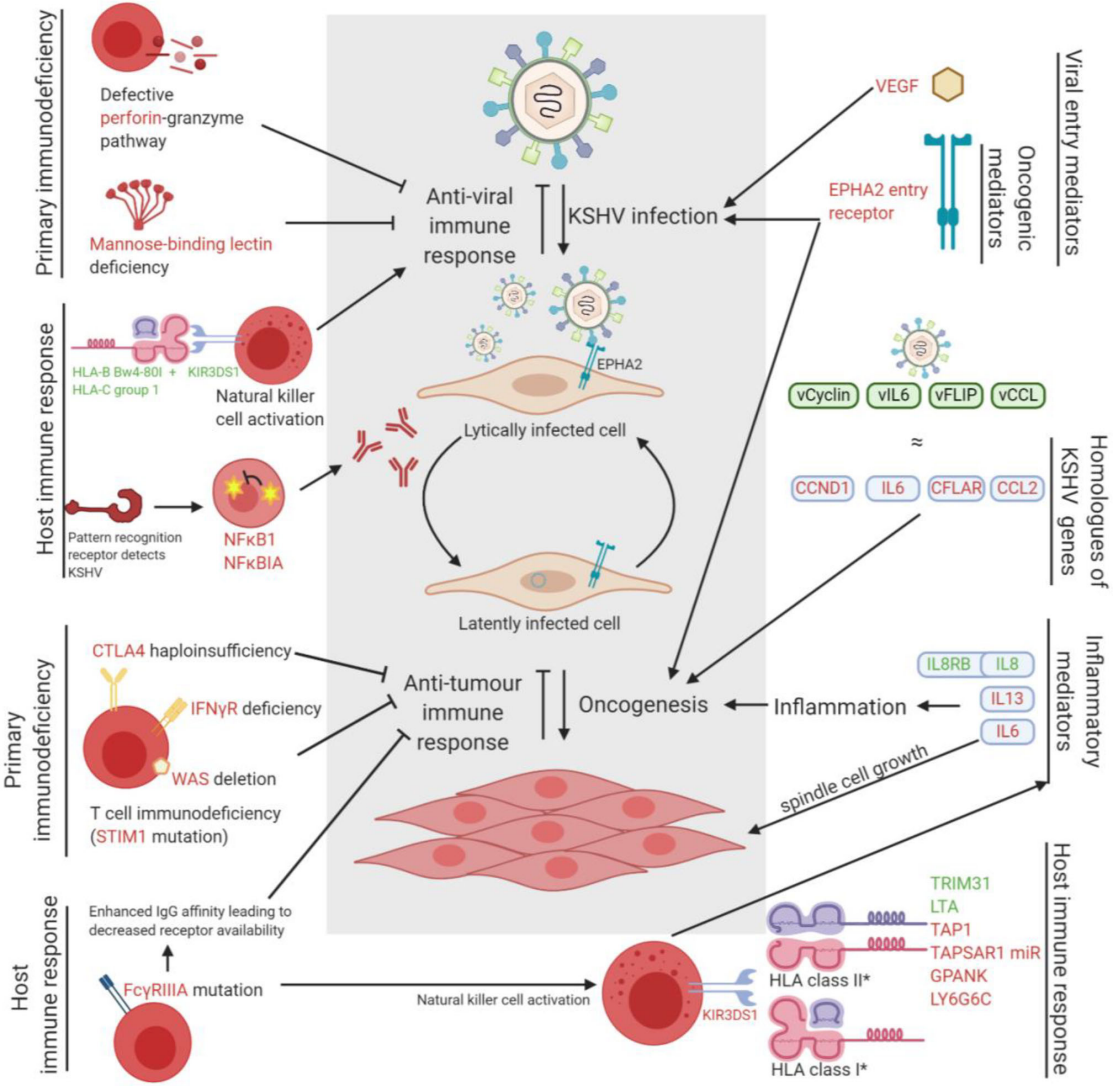
Summary figure depicting the reviewed genetic factors associated with KSHV infection and KS development. KSHV infects endothelial cells and following lytic infection, establishes latency from which reactivation events can occur; KS develops from latently infected endothelial cells (grey box). Gene names in green text indicate an association with decreased risk; red text an association with increased risk. Details of the single nucleotide polymorphism or haplotype involved in these associations are found in Table 1 or in text. *Various HLA haplotypes are either protective or increase risk of KS development as detailed in Table 1. Figure created with BioRender.com

The rationale for an underlying genetic risk of KSHV infection and subsequent KSHV‐associated disease development is well established in the literature through epidemiological observation. The numerous association studies reviewed here point to interesting candidate genes that may prove important in achieving a more nuanced understanding of the pathogenesis and therapeutic targeting of KSHV. Taken together (Figure 1), the studies reviewed here indicate a plausible connection between KSHV susceptibility and host genetic factors that may affect either host immunity to viral infection (perforin‐granzyme pathway, MBL, NK cell activation) or viral access to host cells via entry mechanisms (EPHA2 entry receptor). KSHV‐associated pathogenesis subsequent to infection is likely influenced by an orchestrated concert of innate immune system interactions (NK‐*KIR*‐*HLA*), downstream inflammatory pathways (interleukins, NK cell activation) and oncogenic mechanisms (EPHA2 oncogenic pathways). Infectious diseases and related pathologies are likely to become an increasing global public health burden, particular in parallel with the HIV epidemic and in the era of HAART.^95^ Identifying genetically susceptible populations and affected genes in the pathogenesis of KSHV therefore remains an important scientific goal.

## CONFLICT OF INTEREST

The authors have no competing interest.

## AUTHOR CONTRIBUTIONS

Melissa J. Blumenthal and Georgia Schäfer conceptualised the review. Melissa J. Blumenthal performed the literature search and wrote the manuscript. Georgia Schäfer, Arieh A. Katz, Elena Maria Cornejo Castro and Denise Whitby contributed to the interpretation and critically revised the manuscript. All authors read and approved the final manuscript.

## Data Availability

The data that support the findings of this study are openly available at PubMed (https://pubmed.ncbi.nlm.nih.gov/).
